# The Relationship between Alexithymia and Mental Health Is Fully Mediated by Anxiety and Depression in Patients with Psoriasis

**DOI:** 10.3390/ijerph19063649

**Published:** 2022-03-19

**Authors:** Rossella Mattea Quinto, Francesco De Vincenzo, Dario Graceffa, Claudio Bonifati, Marco Innamorati, Luca Iani

**Affiliations:** 1 Department of Human Sciences, European University of Rome, 00163 Rome, Italy; francesco.devincenzo@unier.it (F.D.V.); marco.innamorati@unier.it (M.I.); 2 Center for the Study and Treatment of Psoriasis, San Gallicano Dermatological Institute, IRCCS, 00144 Rome, Italy; dario.graceffa@ifo.it (D.G.); claudio.bonifati@ifo.it (C.B.)

**Keywords:** dermatology, psychological distress, quality of life, mediation model

## Abstract

Background: Psoriasis is a common skin disease that affects quality of life, especially mental health. Alexithymia has been considered a relevant feature in psoriasis patients. Moreover, psoriasis was found to be associated with negative psychological health, including anxiety and depression. As the pathways linking alexithymia and mental health remain unclear among patients with psoriasis, we aimed to examine the mediating role of anxiety and depression in the relationship between alexithymia and mental health in these patients. Methods: To explore our variables of interest, we used the Toronto Alexithymia Scale (TAS-20), the 12-Item Short Form Health Survey (SF-12), and the Hospital Anxiety and Depression Scale (HADS). Results: Forty-four percent of patients were alexithymic and reported higher anxiety and depression, and lower quality of life compared to non-alexithymic patients. Alexithymic patients also had lower educational attainment. A correlation analysis showed positive associations between alexithymia and both anxiety and depression, whereas mental and physical health were negatively associated with alexithymia. Moreover, anxiety and depression fully mediated the relationship between alexithymia and mental health. Conclusions: Our findings highlight the importance of assessing alexithymia and psychological distress in clinical practice to identify vulnerable patients and to implement interventions aimed at improving negative emotional states.

## 1. Introduction

How and why is alexithymia related to poor mental health in patients with psoriasis? The answer to this question could provide a better understanding of why some patients are more likely than others to have poor mental functioning. Moreover, it would be informative to develop interventions for the promotion of positive mental health despite chronic illness. Health-related quality of life (HRQoL) in psoriasis patients has emerged as an important construct in clinical health psychology [1]. Although the relationship between alexithymia and HRQoL has already been examined [2], whether anxiety and depression would account for the association between alexithymia and HRQoL in patients with psoriasis has not been previously investigated by researchers. Our study addresses this issue, and our main contribution is to test a theoretical model linking alexithymia and mental health, whilst examining the mediating role of anxiety and depression.

Psoriasis is a chronic, autoimmune, inflammatory skin disease significantly affecting patients’ health-related quality of life (HRQoL) [3]. Psoriasis was found to be associated with negative psychological health outcomes, including psychiatric morbidity and suicidal ideation [4], anxiety [5], depression [6], psychological distress [7], emotion dysregulation [8], and cognitive impairment [2]. For example, patients with psoriasis were more likely to exhibit suicidal ideation, attempted suicides, and completed suicides than those without psoriasis [9], although the literature on psoriasis and suicidal ideation and behavior is limited and inconsistent, and the relationship between the two remains unclear [10,11]. Moreover, patients with psoriasis are 1.5 times more likely to experience depressive symptoms [6]. The prevalence of anxiety symptoms (7–48%) is higher in these patients than in healthy controls [5]. Furthermore, patients with psoriasis were also found to be less satisfied with their health; had lower HRQoL in social, psychological, and environmental domains; and reported lower satisfaction with their sex life and physical appearance compared to controls [12]. Psoriasis was also found to be associated with increased risk of cognitive impairment and dementia, with the highest risk in patients with moderate-to-severe psoriasis with suboptimal medical control of the disease [13]. From a neurobiological point of view, pro-inflammatory cytokines have been implicated in the pathogenesis of both depression and psoriasis [14]. Finally, a positive association between the expression of serotonin transporter protein and psoriasis severity was also found, suggesting that serotonin may play a role in the pathophysiology of both depression and psoriasis [15]. In summary, there is an elevated burden of psychiatric comorbidity and impaired quality of life in patients with psoriasis [14].

Alexithymia is a personality trait encompassing a difficulty in identifying and describing feelings as well as an externally oriented thinking [16]. Several studies found a high prevalence of alexithymia in patients with skin diseases, including psoriasis [17,18,19,20]. For example, alexithymic patients with psoriasis had a poorer quality of life, higher anxiety and depression scores, and a higher risk of alcohol dependency compared to patients with less severe alexithymic traits [20]. Moreover, alexithymia was also found to predict psychopathology (e.g., anxiety and depression) [21,22]. However, this trait did not mediate the relationship between psoriasis symptoms and mental HRQoL [8]. Despite the clinical importance of alexithymia in psoriasis, little is known about the underlying mechanisms linking this trait to clinically relevant outcomes.

Many studies have shown positive correlations between alexithymia and measures of anxiety and depression (for a review, see [23]). Moreover, depression was found to partially mediate the relationship between alexithymia and both physical and mental functioning in patients with end-stage organ disease [24]. Individuals with alexithymia have deficits in the ability to link their emotional states to factors eliciting these emotions. As a consequence, alexithymic individuals fail to regulate and cope effectively with negative emotions following stressful events [25], leading to the perpetuation of negative emotions, which may in turn result in anxiety and depression symptoms. In other words, alexithymic individuals are prone to undifferentiated negative affective states [16]; therefore, “the negative affect remains unmodulated, yielding a chronic, yet undifferentiated dysphoria” [25] (p. 52). Moreover, alexithymia has been classified into primary and secondary based on its temporal stability [26]. Primary alexithymia is defined as a stable personality trait and is hypothesized to be causally related to psychological disorders due to impaired emotional processing (i.e., predisposition hypothesis) [27]. Conversely, secondary alexithymia is defined as a state reaction to the effects of a medical illness (i.e., reactivity hypothesis) [26,28].

It is interesting to note that the high prevalence of alexithymia in depressive and anxiety disorders cannot be accounted for by shared variance between alexithymia and these disorders [16]. The close relation between alexithymia and depression is well known. Although they have been described as similar constructs, empirical evidence suggests alexithymia to be distinct from depression despite them being closely related [29]. Indeed, alexithymia is likely to be higher in patients with anxiety or depressive disorders compared to healthy subjects, even after adjusting for confounding variables (e.g., [30]). Whether alexithymia is a predisposing factor or the consequence of depression, or whether they coexist, is still debated; nevertheless, to date, most studies have found support for the predisposition role of alexithymia [29]. As empirical data suggest that alexithymia predisposes individuals to negative emotions, the relationship between alexithymia and health outcomes may be better explained by anxiety and depression.

Although previous studies have examined the mechanisms by which alexithymia is associated with clinically relevant outcomes in different chronic diseases, little is known about these processes in dermatological patients. For example, the difficulty in identifying emotions (i.e., a facet of alexithymia) had an indirect effect on mental health through depressive symptoms in patients with fibromyalgia [31]. Furthermore, depression mediated the relationship between alexithymia and several dimensions of illness perception in patients with systemic lupus erythematosus [32]. Moreover, the difficulty in identifying emotions affected somatization through distress and mental health impairment in patients with chronic pain, supporting the idea that alexithymia may increase somatic symptoms through emotional distress [33]. Finally, depression partially mediated the relationship between alexithymia scores and both physical and mental functioning in candidates for solid organ transplantation [24].

In light of the above considerations, does alexithymia affect mental health via anxiety and depression in patients with psoriasis? Specifically, does alexithymia have positive associations with anxiety and depression which, in turn, negatively affect mental health? The aim of the present study was to investigate whether the relationship between alexithymia and mental health was better explained by anxiety and depression. Consistently, the following hypotheses were tested: (1) alexithymia would affect mental HRQoL and (2) anxiety and depression would serially mediate this relationship. We also aimed to examine the differences between alexithymic and non-alexithymic patients with psoriasis on psychological, clinical, and socio-demographic characteristics.

## 2. Materials and Methods

### 2.1. Sample

For the purpose of this study, we combined datasets from two previous studies [2,8] in which the enrollment was based on the same inclusion/exclusion criteria. The present cross-sectional study involved 150 consecutive outpatients with psoriasis enrolled at the San Gallicano Dermatological Institute of Rome between January 2015 and May 2017. Inclusion criteria were: (a) a diagnosis of psoriasis made by a dermatologist; (b) age ≥ 18 years old. Exclusion criteria were: (a) the presence of major psychiatric disorders (e.g., schizophrenia and bipolar disorder); (b) the presence of current major diseases of the central nervous system (e.g., dementia and Parkinson’s disease) and a history of head injuries or strokes; (c) the presence of specific medical conditions (e.g., hypertension and diabetes mellitus) or other immune-mediated diseases sharing the same physiological mechanism (e.g., rheumatoid arthritis and psoriatic arthritis); and (d) the inability to complete questionnaires. Exclusion criteria were assessed by a board-certified dermatologist through medical records.

### 2.2. Measures

Eligible patients provided information on psychological and sociodemographic (i.e., age, sex, marital status, years of education, and job status) measures. Clinical data, including disease duration, family history of psoriasis, body mass index (BMI), and disease severity, were collected from clinical records.

The Toronto Alexithymia Scale (TAS-20) [34] was used to assess alexithymia. The questionnaire consists of 20 items, measured on a 5-point Likert scale (1 = *strongly disagree*, 5 = *strongly agree*). The TAS-20 measures three dimensions of alexithymia: difficulty in identifying feelings; difficulty in describing feelings; externally-oriented thinking. According to the scoring system, a total score equal to or greater than 61 indicates alexithymia, a score ranging from 52 to 60 suggests borderline alexithymia, and a score equal to or less than 51 indicates the absence of alexithymia. The Cronbach alpha in the present sample was 0.80.

The 12-Item Short Form Health Survey (SF-12) [35] was used to measure health-related quality of life (HRQoL) and general health status. The questionnaire includes eight domains (i.e., physical functioning, role physical, bodily pain, general health, vitality, social functioning, role emotional, and mental health) combined into two scales: a physical component summary (PCS) and a mental component summary (MCS), reflecting physical and mental health, respectively. Higher scores indicate better physical and mental HRQoL. The Cronbach alphas were 0.71 for the PCS and 0.64 for the MCS.

Anxiety and depression were assessed using the Hospital Anxiety and Depression Scale (HADS) [36]. The scale is composed of two subscales measuring anxiety and depression. Items were rated on a 4-point scale indicating the way respondents felt over a 1-week period prior to measurement. Higher scores indicate higher levels of anxiety and depression. In the present sample, Cronbach’s alphas were 0.73 and 0.82 for depression and anxiety, respectively.

The Psoriasis Area Severity Index (PASI) was used to assess disease severity and the extent of psoriasis (in terms of erythema, infiltration, and desquamation) in four body areas (i.e., head, upper extremities, trunk, and lower extremities) [37]. The PASI score ranges from 0 to 72, with higher scores indicating higher disease severity.

### 2.3. Statistical Analysis

All statistical analyses were performed using SPSS (Version 19.0; IBM Corp, Armonk, NY, USA). Differences between alexithymic (i.e., scoring above the cut-off value of 52) and non-alexithymic patients with respect to socio-demographic, clinical, and psychological variables were assessed using a *t*-test for independent samples and a Chi-square test. Pearson’s correlation analysis was conducted to examine the association between psychological (i.e., alexithymia, PCS, MCS, anxiety, and depression) and clinical variables (i.e., disease duration, PASI, and BMI).

To test our hypotheses, we conducted a serial mediation analysis using the PROCESS macro for SPSS v22 [38]. Model 6 of the PROCESS macro was used, with alexithymia as the independent variable, anxiety and depression as serial mediators, and MCS as the dependent variable. A path analysis was used to test the theoretically driven hypothesis that anxiety precedes depression (e.g., [39,40]). The bootstrap estimates were based on 5000 bootstrap samples, with a 95% confidence interval (CI).

All statistics were considered significant if *p* < 0.05. Occasional missing values were imputed by calculating, for each participant, the mean score of the subscale and then replaced.

## 3. Results

### 3.1. Sample Characteristics

The socio-demographic, clinical, and psychological characteristics of the sample are shown in Table 1. Most patients were males, married, and employed. More than half of the patients were overweight or obese. The mean PASI score of patients indicated low disease severity (<10).

### 3.2. Differences between Alexithymic and Non-Alexithymic Patients

Table 1 shows the comparisons of socio-demographic and clinical features between alexithymic and non-alexithymic patients with psoriasis. Forty-four percent of patients reported borderline or high alexithymia scores. Patients with borderline to high alexithymia reported higher levels of anxiety (*p* < 0.001) and depression (*p* < 0.001), as well as lower PCS (*p* = 0.006) and MCS (*p* = 0.039) scores when compared to non-alexithymic patients. Furthermore, about 70% of patients in the borderline/high alexithymia group had low levels of education (i.e., equal to or less than 8 years; data not shown). Furthermore, years of education was the only socio-demographic variable that was significantly lower in patients with borderline to high alexithymia compared to non-alexithymic patients (*p* < 0.001). Finally, there were no between-groups differences in clinical variables, including BMI (*p* = 0.268), PASI (*p* = 0.653), family history of psoriasis (*p* = 0.109), and disease duration (*p* = 0.315).

### 3.3. Correlations between Variables

The associations between variables are reported in Table 2. Alexithymia was positively and moderately associated with both anxiety (*p* < 0.001) and depression (*p* < 0.001). PCS and MCS scores were negatively associated with alexithymia (*p* = 0.005 and *p* = 0.036, respectively), as well as with anxiety (*p* = 0.003 and *p* < 0.001, respectively) and depression (*p* < 0.001 and *p* < 0.001, respectively). No significant correlations were found between alexithymia and other clinical variables, including disease duration, PASI, and BMI. Finally, disease severity was negatively associated with PCS (*p* = 0.027), whereas no significant associations emerged between PASI and other psychological variables.

### 3.4. Serial Mediation Analysis

**Figure 1:**
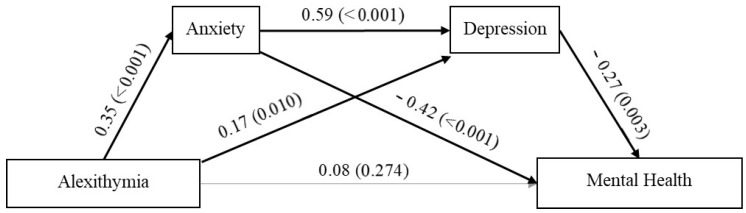
The mediating role of anxiety and depression in the relationship between alexithymia and mental health (β, *p*). Bold lines indicate significant mediation. Standardized coefficients are reported.

Figure 1 shows direct effects between the variables included in the present serial mediation model. The mediation analysis showed that alexithymia affected mental health through different pathways, but direct effects were not significant (β = 0.08, SE = 0.05; 95% CI (−0.046, 0.161)). Alexithymia predicted MCS via anxiety (β = −0.15, SE = 0.04; 95% CI (−0.246, −0.071)) and depression (β = −0.05, SE = 0.02; 95% CI (−0.103, −0.007)). There was also a significant serial indirect effect of alexithymia on mental health through anxiety and depression (β = −0.06, SE = 0.02; 95% CI (−0.109, −0.019)), indicating that alexithymic patients are more likely to report lower levels of mental health through the experience of high levels of anxiety and depression. We then conducted pairwise comparisons among the three indirect effects to compare the strengths of these associations. Specific indirect effects did not significantly differ in magnitude.

## 4. Discussion

In this study, we examined the associations between alexithymia and mental health, and tested a model in which this relation was serially mediated by anxiety and depression. Our findings indicate that the relationship of alexithymia with mental health is fully mediated by anxiety and depression. In our sample, the prevalence of alexithymic traits is 44%. This finding is consistent with those of previous studies showing a significant association between alexithymia and psoriasis, e.g., [41,42]. In particular, Innamorati et al. [8] found that the majority of patients reported borderline to high levels of alexithymia. Notwithstanding the consistency of these results, the underlying pathways linking alexithymia and mental health have not yet been investigated. Our mediation analysis shows that alexithymia influences mental health only through the presence of negative emotional conditions (i.e., anxiety and depression). Significant indirect effects and the theoretically driven hypothesis [39,40] justify considering anxiety and depression in a causal order in the serial mediation model. Our results show that alexithymia influences MCS through three pathways: (a) the separate mediation of anxiety; (b) the separate mediation of depression; (c) the serial mediation of anxiety and depression. Moreover, pairwise comparisons show that indirect effects do not differ significantly from each other in terms of magnitude. This finding indicates that the mediating role of anxiety is not significantly different from those of depression and anxiety and depression in serial. It is likely that alexithymia impairs the skills that regulate and cope with the negative emotions following the stressful event of the chronic illness and its sequelae. According to previous theoretical studies [16,25], we hypothesize that alexithymia predisposes patients with psoriasis to a negative emotional valence, which in turn negatively affects mental functioning. Therefore, negative emotions as a consequence of alexithymic traits may be one of the key mechanisms which explains lowered mental health in patients with psoriasis. Our results are also consistent with the predisposition hypothesis, which posits that alexithymia is a stable personality trait (i.e., primary alexithymia) and is causally related to psychopathology [26,27]. As suggested by previous studies (e.g., [24]), patients with higher levels of alexithymia who report poor mental functioning may be at an increased risk of depression.

However, it is also possible that high levels of either alexithymia or depression/anxiety may reflect a reaction to stressful situations (e.g., the presence of psoriasis symptoms), which may in turn impact mental health (i.e., reactivity hypothesis). As the present study used a cross-sectional design, our results do not rule out this alternative explanation. Although prospective have studies found that alexithymia showed a high level of relative stability in spite of acute increases in psychological distress (e.g., [43]), the predisposition and reactivity hypotheses are still a matter of debate [29]. Hence, future longitudinal studies should examine both the temporal stability and state dependence of alexithymia to determine the nature of the relationship between alexithymia and psychological distress in patients with psoriasis.

In the present study, we also examined differences between alexithymic and non-alexithymic psoriasis patients in psychological variables. Alexithymic patients reported higher levels of anxiety and depression, as well as lower levels of physical and mental HRQoL as compared to non-alexithymic patients. Previous studies have shown that alexithymia predicted both anxiety and depression in patients with psoriasis (e.g., [21,22]), although alexithymic patients reported higher levels of anxiety, but not depression, than non-alexithymic patients [22]. The different results in depression scores for alexithymic patients may be due to differences in the TAS-20 cut-off (a total score above 49 was used to indicate alexithymia in the study of Korkoliakou et al.). In our sample, alexithymic and non-alexithymic patients do not differ in clinical features (e.g., PASI, BMI, and disease duration). This result is in line with those of previous studies [22,44,45]. Nevertheless, we examined patients with mild-to-moderate disease severity; it is likely that an association between alexithymia and clinical features exists in more severe conditions, increasing the need for illness adaptation. Moreover, alexithymic patients with psoriasis also have a lower level of school education in comparison with non-alexithymic patients. Similarly, Cherrez-Ojeda et al. [17] found the highest education level to predict lower alexithymia in patients with psoriasis. This result is consistent with those of previous studies (e.g., [46,47]), which hypothesized the presence of an educational bias; higher education is linked to better expressive skills, whereas lower education is related to less understanding of emotional states and disclosure.

The results of the present study should be interpreted in light of the following limitations. First, the cross-sectional design does not allow to draw causal conclusions. Second, in our study the majority of patients had mild psoriasis. Future studies should include patients with severe psoriasis, to examine these relationships according to disease severity. Third, we used self-report measures which are potentially biased by social desirability; future studies could use a multimodal assessment approach to reduce risk of bias. Fourth, we did not include the TAS-20 subscales in mediation analysis due to poor reliability. Future studies should consider alexithymia facets to better clarify which difficulties in experiencing and regulating affects cognitively are more relevant for HRQoL impairment in patients with psoriasis.

## 5. Conclusions

Notwithstanding these limitations, this study is among the first to examine the mediating role of anxiety and depression in the relationship between alexithymic traits and mental health among psoriasis patients. Based on the results of the present study, it is important that dermatologists include the assessment of alexithymia and psychological distress in clinical practice to identify vulnerable patients and to implement interventions aimed at improving negative emotional states. This, in turn, will reduce the risk of HRQoL impairment in patients with psoriasis high in alexithymia. Despite the high comorbidity of psychiatric disorders in patients with psoriasis, there are few recommendations on how clinicians should identify and address mental health issues in their patients [14]. Routine screening of anxiety and depression, namely, the most common mental health problems in psoriasis, is recommended. Clinical and health psychologists may help in the assessment and treatment of these psychological disorders. Clinicians should also assess potential risk factors for increased suicidal behaviors (i.e., combined attempted and completed suicides). If patients have severe psychiatric symptoms or concerns regarding suicidal ideation or attempted suicides, a consultation with a psychiatrist is necessary [14].

Our findings pave the way for further investigations on how psychological interventions could improve mental health by treating negative emotional states and psychopathology. For example, mindfulness-based interventions, characterized by exercises to enhance attention in the present moment, awareness of bodily sensations, and non-judging in observing thoughts and feelings, are effective in improving alexithymia, and other measures of psychological health (e.g., worry, anxiety, and depression) (for systematic reviews, see [48,49]). Moreover, skills-based interventions (i.e., Cognitive Behavioral Therapies) may be particularly suitable for patients high with alexithymia (e.g., [50]), who are guided to describe their feelings and thoughts experienced during exposure exercises, thereby improving emotional awareness and the ability to identify, describe, regulate, and cope with negative emotional states. As alexithymia and psychopathology significantly affect mental health among psoriasis patients, future studies may further investigate how psychological interventions (e.g., mindfulness and CBT) could reduce HRQoL impairment by improving emotional awareness and positive emotional states in patients with psoriasis.

## Figures and Tables

**Table 1:** Comparisons of socio-demographic and clinical features between alexithymic (positive or borderline positive) and non-alexithymic psoriasis patients.

Variable, *M* (*SD*)		No	Borderline/High	Total	*t* or χ^2^	*p*
		Alexithymia	Alexithymia	Sample		
		(*N* = 84)	(*N* = 66)	(*N* = 150)		
Age		45.94 (13.83)	49.80 (15.79)	47.64 (14.80)	*t*(129.9) = −1.57	0.119
Sex, *N* (%)						0.566
	Male	47 (54.0)	40 (46.0)	87 (58.0)		
	Female	37 (58.7)	26 (41.3)	63 (42.0)		
Marital status, *N* (%)					χ^2^(1, *N* = 150) = 0.15	0.928
	Not married	26 (55.3)	21 (44.7)	47 (31.3)		
	Married	46 (55.4)	37 (44.6)	83 (55.4)		
	Divorced/widowed	12 (60.0)	8 (40.0)	20 (13.3)		
Years of education		13.44 (3.12)	11.23 (3.53)	12.47 (3.47)	*t*(130.7) = 4.01	<0.001
Job status, *N* (%)					χ^2^ (1, *N* = 150) = 1.74	0.627
	Not employed	13 (59.1)	9 (40.9)	22 (14.7)		
	Employed	59 (57.8)	43 (42.2)	102 (68.0)		
	Retired	7 (41.2)	10 (58.8)	17 (11.3)		
	Student	5 (55.6)	4 (44.4)	9 (6.0)		
Disease duration		17.10 (14.08)	19.36 (12.84)	18.10 (13.55)	*t*(140.0) = −1.00	0.315
PASI		4.31 (2.89)	4.11 (2.67)	4.22 (2.79)	*t*(144.2) = 0.45	0.653
Family history of psoriasis, *N* (%)		38 (45.2)	38 (58.5)		χ^2^ (1, *N* = 150) = 2.56	0.109
BMI					χ^2^ (1, *N* = 150) = 2.63	0.268
	BMI ≤ 25	35 (59.3)	24 (40.7)	59 (39.3)		
	BMI 25.1–29.9	32 (60.4)	21 (39.6)	53 (35.4)		
	BMI ≥ 30	17 (44.7)	21 (55.3)	38 (25.3)		
Anxiety		5.46 (3.05)	7.65 (4.10)	6.43 (3.70)	*t*(116.5) = −3.62	<0.001
Depression		4.69 (3.29)	7.20 (3.90)	5.79 (3.77)	*t*(126.9) = −4.18	<0.001
PCS		50.22 (6.84)	46.61 (8.63)	48.62 (7.87)	*t*(122.1) = 2.77	0.006
MCS		43.26 (9.69)	40.06 (8.97)	41.84 (9.48)	*t*(143.6) = 2.08	0.039

PASI = Psoriasis Area Severity Index; BMI = body mass index; PCS = SF-12 physical component summary; MCS = SF-12 mental component summary.

**Table 2:** Relationships between variables in the whole sample.

	1	2	3	4	5	6	7
1 Disease duration	1						
2 PASI	0.13						
3 BMI	0.28 **	0.17 *					
4 Anxiety	0.05	0.04	0.04				
5 Depression	0.05	0.05	0.09	0.65 ***			
6 PCS	−0.17 *	−0.18 *	−0.28 **	−0.24 **	−0.32 ***		
7 MCS	0.06	−0.12	-0.01	−0.57 ***	−0.51 ***	−0.10	
8 TAS-Tot	0.16	0.03	0.11	0.36 ***	0.38 ***	−0.23 **	−0.17 *

* *p* < 0.05; ** *p* < 0.01; *** *p* < 0.001. PASI = Psoriasis Area Severity Index; BMI = body mass index; PCS = SF-12 physical component summary; MCS = SF-12 mental component summary; TAS = Tot = Toronto Alexithymia Scale (total score).

## Data Availability

The data presented in this study are available on request from the corresponding authors (R.M.Q. and L.I.). The data are not publicly available due to privacy or ethical restrictions.
